# One-Step Dry-Etching Fabrication of Tunable Two-Hierarchical Nanostructures

**DOI:** 10.3390/mi15091160

**Published:** 2024-09-17

**Authors:** Xu Ji, Bo Wang, Zhongshan Zhang, Yuan Xiang, Haifang Yang, Ruhao Pan, Junjie Li

**Affiliations:** 1 Beijing National Laboratory for Condensed Matter Physics, Institute of Physics, Chinese Academy of Sciences, Beijing 100190, China; jixu@iphy.ac.cn (X.J.); wangbo2014@iphy.ac.cn (B.W.); zhangzs@iphy.ac.cn (Z.Z.); xiangyuan@iphy.ac.cn (Y.X.); hfyang@iphy.ac.cn (H.Y.); 2 CAS Key Laboratory of Vacuum Physics, University of Chinese Academy of Sciences, Beijing 100049, China

**Keywords:** two-hierarchical nanostructures, nanofabrication platform, one-step etching, inductively coupled plasma reactive ion etching, high controllable degrees

## Abstract

Two-hierarchical nanostructures, characterized by two distinct configurations along the height direction, exhibit immense potential for applications in various fields due to their significantly enhanced controllable degree compared to single-order structures. However, due to the limitations imposed by planar technology, the realization of two-hierarchical nanostructures encounters huge challenges. In this work, we developed a one-step etching method based on inductively coupled plasma reactive ion etching for two-hierarchical nanostructures. Thanks to the shrinking effect of the Cr mask and the generation of a passivation layer during etching, the target materials experienced two different states from vertical etching to shrink etching. Consequently, the achieved two-hierarchical nanostructure configuration features a cross-section of an upper triangle and a lower rectangle, showing higher controllable degrees compared to the one-order ones. Both the mask pattern and etching parameters play crucial roles, by which two-hierarchical structures with diversiform shapes can be constructed controllably. This method for two-hierarchical nanostructures offers advantages including excellent control over structural properties, high processing efficiency, uniformity across large areas, and universality in materials. This developed strategy not only presents a simple and rapid nanofabrication platform for realizing optoelectronic devices, but also provides innovative ideas for designing the next generation of high-performance devices.

## 1. Introduction

Compared to nanostructures, two-hierarchical nanostructures, which show two distinct shapes along the cross-section of the height direction, possess much higher controllable degrees of freedom and thus can be used in a series of high-performance devices, including optical metasurfaces, anti-reflection elements, photon emitters, and probes, and thus pave a fresh strategy for the next generation of optoelectronic and measurement instruments. For instance, anti-reflection devices can suppress the reflected light signal of a surface, and can be potentially applied in radars, optical invisible cloaks, solar cells, etc. [1,2]. Additionally, anti-reflection devices that consist of two-hierarchical nanostructures can realize higher efficiency and a wider operating band, showing significant advancements in many fields [3,4,5]. Meanwhile, the photon emitter that relies on the two-hierarchical nanostructure configuration can be used in quantum devices, field emission cathodes, and other important devices [6,7]. Despite the advantages, the difficulties in fabrication severely limit the practical application of two-hierarchical nanostructures, because the configuration shift along the z-axis is beyond the capacity of traditional planar art. Thus, a new fabrication method for two-hierarchical nanostructure blocks at the nanoscale is urgently needed for the further development of next-generation micro-/nano-devices.

The great advantages of two-hierarchical nanostructures have attracted the interest of researchers, and they focus on the fabrication of the nanostructures and have developed many techniques. These methods concentrate on solving the problems faced by fabricating two-hierarchical nanostructures, such as large-area construction, nanoscale fabrication, and high spatial controllability. The direct 3D fabrication methods were first considered, and these methods provide a method for 3D structures with arbitrary shapes. However, the corresponding method cannot achieve two-hierarchical nanostructures with high precision and a large area, and the 3D print method has no capacity to process structures at the nanoscale. Three-dimensional laser direct writing based on two-photon absorption can be applied in the nanostructures of several hundred nanometers, but the applicable materials and processing speed do not meet the need of practical devices [8,9,10]. Focused ion beam (FIB)-induced deposition is a special 3D printing method that can build up structures at the nanoscale, but this approach cannot be used in large-area preparations because its point-to-point fabrication regime results in a very low fabricating efficiency [11,12,13]. Nowadays, two-hierarchical nanostructures are mostly fabricated by adjusting the planar methods, which include lithography, deposition, and etching. Wet etching is a process in which a sample is immersed in a certain chemical reagent, and the materials not covered by the mask can be removed through a chemical reaction. Complex 3D structures can be achieved through wet etching, but there are disadvantages, such as the slow etching rate, poor spatial controllability, and serious chemical pollution [14,15,16]. The templated electrochemical deposition (ECD) method is also suitable for the fabrication of two-hierarchical nanostructures based on the overgrowth effect. However, this method can only be used to fabricate metallic configurations, and the need of a conductive layer limits the fabrication of structures on dielectric substrates [17]. Thus, a one-step, high-productivity method for the fabrication of two-hierarchical nanostructures with great precision is urgently needed to overcome the bottlenecks of the other relative methods and push the applications of the two-hierarchical configurations to a new stage.

Here, we developed and experimentally demonstrated a two-hierarchical nanostructure fabrication method based on one-step inductively coupled plasma (ICP) reactive ion etching. Due to the mask deformation and the generation of a passivation layer during the etching process, two distinct kinds of etching phenomenon, namely, vertical etching and shrink etching, can be observed, and thus the output structures show a cross-section with an upper triangle and a bottom rectangle. Two-hierarchical nanostructures built by this technology show arbitrary patterns such as nanogratings and polygons by adjusting mask patterns and etching parameters. This method has a simple processing flow and all the technologies used in this approach meet the needs of large-area preparation. The minimum distance between structures can reach 10 nm, and the minimum cone radius of triangles can also reach 10 nm. Additionally, the structure height, linewidth, period, and other parameters can be tuned within a large range, with one-step molding, and no complex processing steps are required. The two-hierarchical nanostructure processing method in this experiment can achieve a breakthrough in dimensions and provide new ideas for the research and development of nanodevices in optical and electrical fields.

## 2. Methods

Firstly, TiO_2_ with a thickness of 800 nm was deposited on a silicon wafer by electron beam deposition (EBD, FU-12PEB-500, Taiwan, China). Then, Cr with a thickness of 60 nm was deposited on TiO_2_ by EBD as the mask layer with a thickness precision of 3%. After that, an e-beam resist (AR-P 6200.13, Berlin, Germany) was spin-coated on the wafer and baked at 150 °C for 1 min to obtain a 300 nm thick film. The resist was prepatterned by electron beam lithography (EBL, Raith EBPG 5200, Dortmund, Germany) and developed by immersing it in the developer (AR-600-546, Berlin, Germany) for 1 min and the stopper (DI water) for 30 s, and thus, the resist mask was obtained. The Cr mask was then achieved by the pattern transfer process utilizing an ICP (LEUVEN Heverlee Poshow A) system. The etching parameters were set to an ICP power of 400 W and RF power of 40 W; the gas flows were fixed at Cl_2_ 80 sccm and O_2_ 20 sccm; and the chamber pressure was set to 10 mTorr. The residual photoresist was then removed using RIE. Finally, the TiO_2_ was etched using ICP, which can directly etch two-hierarchical nanostructures with a triangular upper layer and a rectangular lower layer. The process parameters were ICP power of 350 W and RF power of 120 W; the gas flows were fixed at SF_6_ 40 sccm, CHF_3_ 10 sccm, and O_2_ 5 sccm; and the chamber pressure was set to 10 mTorr. The TiO_2_ was then etched under a temperature of 40 °C. All the gases with a purity of 99.999% used in this work were purchased from Minxing Gas Ltd., Maoming, China.

## 3. Results and Discussion

**Figure 1:**
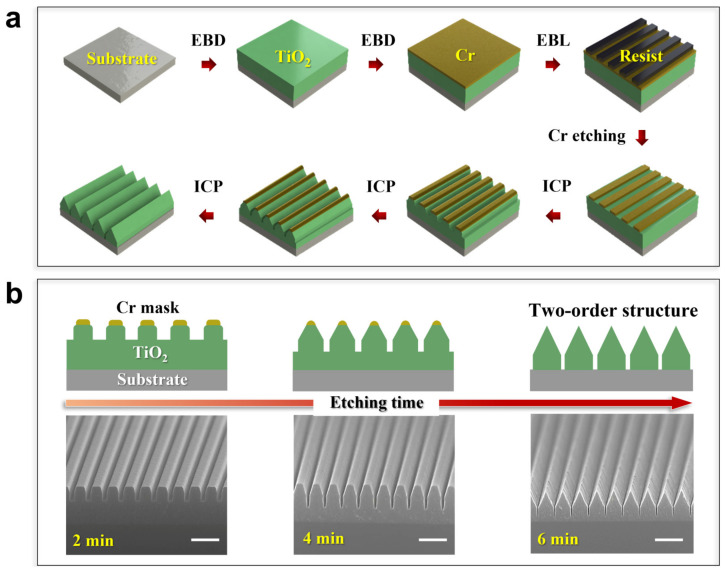
(**a**) Process flow diagram for two-hierarchical nanostructure preparation. (**b**) With increasing time, the evolution of the morphology of this two-hierarchical nanostructure. Scale bar: 600 nm.

**Figure 2:**
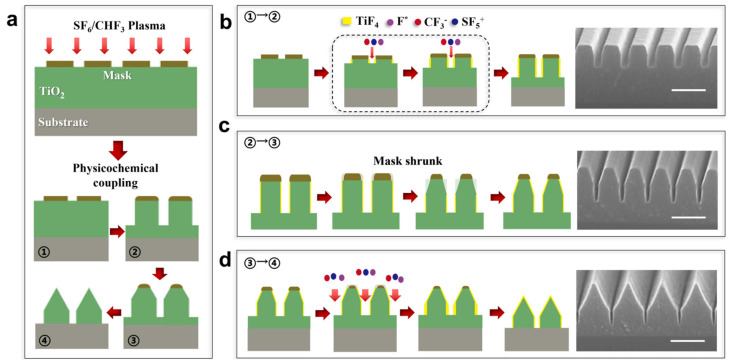
One-step etching fabricating mechanism of two-hierarchical nanostructures. (**a**) The process of two-hierarchical nanostructure formation through the action of CHF_3_ and SF_6_ plasma in the etching process. (**b**) At the beginning of etching, TiO_2_ is etched into vertical gratings. (**c**) With the increase in etching time, the Cr mask gradually narrows due to shrink etching, and the TiO_2_ gradually forms two-hierarchical nanostructures with a trapezoid on the top and rectangle on the bottom. (**d**) When the Cr mask shrink etching is completed, TiO_2_ two-hierarchical nanostructures with a triangular upper section and a rectangular lower section are formed. Scale bar: 400 nm.

The fabrication of the two-hierarchical nanostructures is schematically shown in Figure 1a. A clean Si wafer is chosen for the substrate, and 800 nm TiO_2_ is deposited on the substrate. Here, TiO_2_ is selected for its excellent optical properties. A Cr layer with a thickness of 60 nm is further deposited as the metallic mask. Then, the mask is patterned by the EBL and ICP. The two-hierarchical nanostructures then take the shape followed by the ICP. In this process, the two-hierarchical nanostructures can be achieved by a one-step etching at the nanoscale for shrinking of the mask and the generation of the passivation layer. Namely, the target materials experienced two different states, which are vertical etching and shrink etching, respectively. Consequently, the achieved two-hierarchical nanostructure configuration features a cross-section of an upper triangle and a lower rectangle. Figure 1b clearly shows the shape evolution of the TiO_2_ nanostructures during the ICP etching. A nanograting with a 300 nm pitch and 200 nm critical dimension (CD) is chosen as the test structure. By optimizing the etching parameters, at the start of the etching, the vertical etching takes dominance, and the edges of the mask become a rounder corner, as the left column depicts. With the increase in time, the shrink etching at the top and the passivation layer generation at the bottom can be observed. The TiO_2_ nanograting begins to show a two-hierarchical morphology, where the top pattern is a trapezoid while the bottom pattern is a rectangle (middle column of Figure 1b). The shrink etching comes from the linewidth decrease of the Cr mask, and the passivation layer results in a narrower gap between the stripes. With continuous etching, as the right column demonstrates, the mask is finally removed, and the two-hierarchical configurations are built up with a triangle and a rectangle shape cross-section. In order to reveal the basic mechanism of the one-step etching in constructing two-hierarchical nanostructures, the etching process is investigated in detail and depicted in Figure 2. There are three phenomena that can be found, namely, dissociation, passivation, and sputtering, in which dissociation refers to the reactive gas transfer to radicals:

(1) $$e^{-}+{SF}_{6}\to F^{*}+{{SF}_{5}}^{*}+e^{-}$$

(2) $$e^{-}+O_{2}\to 2O^{*}$$

(3) $$e^{-}+{CHF}_{3}\to H^{*}+{{CF}_{3}}^{*}+e^{-}$$

where *SF*_6_, *O*_2_, and *CHF*_3_ are reactive gases, and F*, *SF*_5_*, O*, *CF*_3_*, and H* are radicals.

The passivation process can be defined as:

(4) $${TiO}_{2}+4F^{*}+4H^{*}\to {TiF}_{4}+2H_{2}O$$

In this process, *TiF*_4_, which is a type of stable solid, is generated as the passivation layer that can protect the nanogratings from being etched. Finally, the sputtering process is as follows:

(5) $$e^{-}+{SF}_{6}\to F^{*}+{{SF}_{5}}^{+}+e^{-}$$

(6) $$e^{-}+{CHF}_{3}\to H^{+}+{{CF}_{3}}^{-}+e^{-}$$

*SF*_5_^+^ and *CF*_3_^−^ refer to different kinds of ion that can be used for the sputtering of TiO_2_.

Figure 2 shows the mechanism of the two-hierarchical nanostructure etching process. Figure 2a shows the process of two-hierarchical nanostructure formation through the action of CHF_3_ and SF_6_ plasma in the etching process. As shown in Figure 2b, at the beginning of etching, TiO_2_ is etched into a vertical grating. The surface of TiO_2_ reacts with F* radicals to form a TiF_4_ passivation layer. As the equations show, the reactive gas is dissociated to kinds of fluoro-based radicals (Equations (1)–(3)). When radicals randomly impact the surface of TiO_2_, the material reacts with F* radicals to form a TiF_4_ passivation layer on the surface, including the side wall and bottom of the nanostructures (Equation (4)). The chemical properties of TiF_4_ are stable and it is more difficult to remove compared to TiO_2_. As for the sputtering, as depicted in Equations (5) and (6), SF_5_^+^ and CF_3_^−^ are generated and etch both TiO_2_ and TiF_4_ along the z-axis, simultaneously, while the generated TiF_4_ on the side wall is retained, which results in the gap between the nanoribbon becoming narrower with the etching time. Furthermore, the shape deformation of the mask plays another key role in the construction of two-hierarchical nanostructures. One can clearly see from Figure 2c that the linewidth of the Cr mask becomes smaller due to the lateral etching of the plasma when the etching depth of the TiO_2_ reaches 300 nm. Consequently, a shrink etching effect can be observed, and the shape of the grating shows a narrower linewidth at the top. When the passivation layer generation and the mask deformation happen synchronously in this etching step, the height of the grating is higher and the gap between structures becomes smaller for the continuous etching along the z-axis and the passivation layer generation, but the top of the nanograting demonstrates a trapezoid shape due to the mask deformation. When the Cr mask shrink etching is completed, as shown in Figure 2d, two-hierarchical nanostructures with an upper section of a triangle and a lower section of a rectangle are finally built up. 

**Figure 3:**
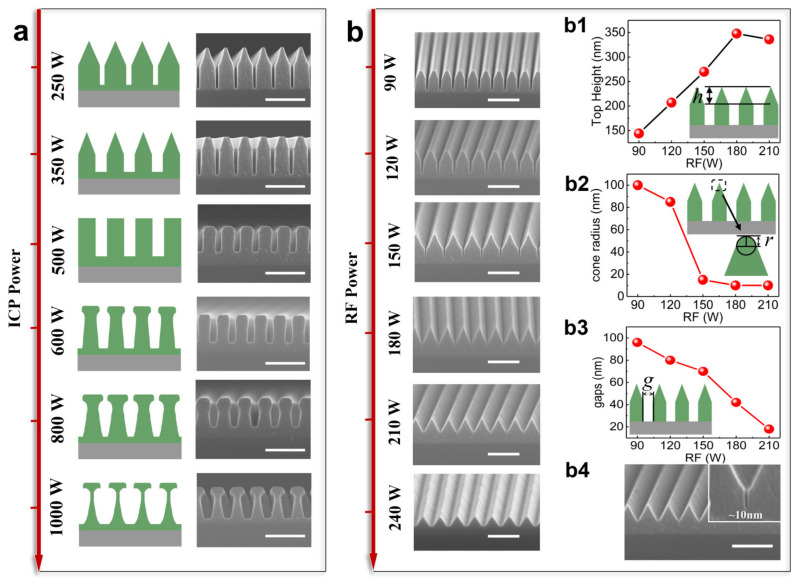
(**a**) Under the condition that only ICP power changes and the other etching conditions remain unchanged, the morphology of the two-hierarchical nanostructures after etching of the same etching mask is affected. (**b**) Under the condition that only RF power changes and the other etching conditions remain unchanged, the morphology of the two-hierarchical nanostructures after etching of the same etching mask is affected: (**b1**–**b3**) the change in the main structural parameters of the two-hierarchical nanostructure morphology when RF power varies, including the height of the upper triangle (h), the radius of the top corner of the triangle (r), and the gap between the structures (g); (**b4**) the minimum gap between cell structures is 10 nm. Scale bar: 600 nm.

After the physical mechanism of fabricating two-hierarchical nanostructures is revealed, the etching parameters are investigated with the purpose of modulating the configuration of the two-hierarchical nanograting. Here, to ensure the gases react adequately, the ratio of the reactive gas flows is firstly optimized via an experiment. The basic etching parameters are ICP power of 350 W and RF power of 120 W; the gas flows are fixed at SF_6_ 40 sccm, CHF_3_ 10 sccm, and O_2_ 5 sccm; the chamber pressure is set to 10 mTorr; and the TiO_2_ is then etched under a temperature 40 °C. Firstly, the ICP power versus the morphology of the nanograting is obtained and shown in Figure 3a, where six samples are etched under the same etching conditions but with different ICP power levels. With the ICP power increasing from 250 W to 1000 W, the transverse etching at the side wall of the gratings becomes obvious as the ICP power gradually increases because the plasma concentration increases due to the large ICP power. When the TiO_2_ is etched under a low ICP power, the cross-section of the nanograting shows a triangle top pattern and a rectangle bottom pattern, and the change in the triangle decreases with the gradual increase in the ICP power. As the ICP power reaches 500 W, the nanostructures become a vertical grating. With the power continuing to increase, the plasma concentration is denser, the top pattern of the grating shows a rectangle shape, and the bottom one is a trapezoid, indicating that one can flexibly tune the top and bottom sections by the ICP power. We further concentrate on the structures obtained by an ICP power of 350 W, but change the RF power from 90 W to 240 W. As Figure 3b shows, as the RF power increases, the gap between the gratings becomes smaller. During this process, more TiF_4_ is sputtered out and accumulates at the bottom, and as the TiF_4_ increases, the position where the plasma etching narrows, so the spacing between the cell structures narrows. Figure 3(b1–b3) show the change in the basic parameters, which include the height of the triangle of the upper section, the radius of the top corner of the triangle, and the gap between the structures of the two-hierarchical nanostructure morphology with the RF. Figure 3(b1) shows the height of the top triangle versus the RF power, indicating that the height increases with the RF power, reaches a maximum of 350 nm under a 180 W RF power, and becomes stable with the continuous increase in the power. The radius of the top corner of the triangle is demonstrated in Figure 3(b2). A decreasing trend is observed with the RF power, meaning that the top edge of the nanostructures becomes sharper with the RF power. The minimum cone radius is 10 nm. Notably, the generated rate of TiF_4_, which defines the gap between the structures, is a function of the RF power. A higher RF power usually results in a thicker passivation layer and results in a decrease in the gap width (Figure 3(b3)), and a minimum gap of 10 nm can be obtained for the RF with a power of 210 W (Figure 3(b4)). The gap finally vanishes with the RF increasing to 240 W, with only the upper triangular structure being left and forming a blazed grating. Thanks to the etching temperature, the reactive gas and power are precisely controlled during the etching process, resulting in a negligible error of the constructed structures.

**Figure 4:**
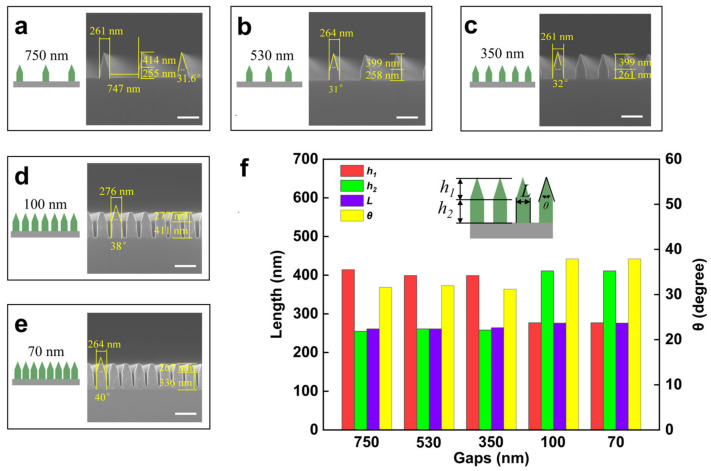
With the same design linewidth, but a different spacing mask, the morphology of the two-hierarchical nanostructures changes under the same etching conditions. (**a**–**e**) Structural features and key parameters of two-hierarchical nanostructures marked on the SEM image. (**f**) Variation trend of key structural parameters with the structural gap regulation, namely, height of the upper triangle (h_1_), height of the lower rectangle (h_2_), angle of the upper triangle (θ), and width (L). Scale bar: 600 nm.

The impact of the gap between the two-hierarchical structures on the morphology is further investigated. A series of two-hierarchical nanostructures with a consistent CD of 200 nm, with gaps of 750 nm, 530 nm, 350 nm, 100 nm, and 70 nm, are designed and fabricated. Figure 4a–e summarize the key parameters of the nanostructures on the scanning electron microscopy (SEM) image, i.e., the height of the upper triangle (h_1_) and the lower rectangle (h_2_), the angle of the upper triangle (θ), and the width (L). The results are the counts shown in Figure 4f. When the gaps are greater than 100 nm, with the gaps decreasing, h_1_, h_2_, θ, and L remain stable. It can be concluded that when the gaps exceed 100 nm, changes in the gaps have no effect on the two-hierarchical nanostructures’ morphology. However, when the gaps are smaller than 100 nm, h_1_, h_2_, and θ have large changes, and only L remains stable. At this time, the gaps have a greater impact on the preparation of the two-hierarchical nanostructures, because the gaps between the unit structures are too small, resulting in a smaller etching area during the etching process, thus causing the etching rate to become faster. When the etching reaches a similar depth, the triangular structure at the upper end of the two-hierarchical nanostructures has not been etched to a sharp cone, leading to a decrease in h_1_, and an increase in h_2_ and θ. The unchanged value of L indicates that the gaps of the two-hierarchical nanostructures in this etching process have no effect on the width of the unit structure. The above-mentioned laws enable one to fabricate two-hierarchical nanostructures on demand, which is very helpful for the design of devices with two-hierarchical nanostructures.

**Figure 5:**
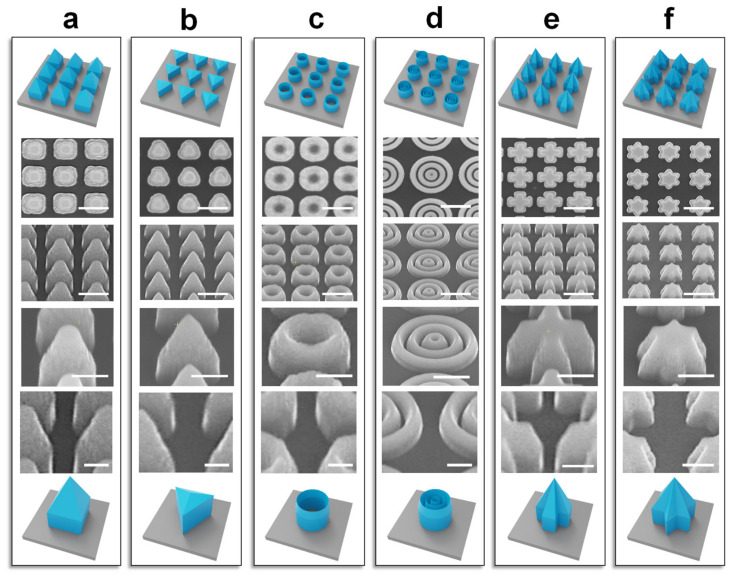
One-step etching of diversified two-hierarchical nanostructures using different masks. From top to the bottom are the schematic, top, and tilt views of an array of two-hierarchical nanostructures, the detailed SEM images of a single nanostructure and the side walls, and finally a schematic of a unit cell of two-hierarchical nanostructures, where the mask shapes are designed as (**a**–**f**) squares, triangles, circles, multi-circles, crosses, and hexagons. Scale bar: 500 nm (second and third rows), 300 nm (fourth row), and 200 nm (fifth row).

The etching of two-hierarchical nanostructures is independent of the direction of the mask, making it possible to build up nano-configurations with diversiform shapes. The etching process was extended from gratings to more intricate array structures. We designed a series of planar patterns to verify the manufacturing capabilities of structures with a sufficient spatial degree. Figure 5a–f show the design drawings of squares, triangles, circles, multi-circles, crosses, and hexagons, and their SEM images after etching, where the structures show great consistency between each other. All the structures show a sharp top layer and a bottom layer with steep side walls, indicating that the method has the feasibility of processing more complex three-dimensional structures. Furthermore, the enlarged views of the side walls of different shapes indicate a smooth surface, showing the high-quality etching capacity of the developed method. This process not only presents a novel fabrication platform for realizing optoelectronic devices at the nanoscale, but also provides innovative ideas for designing the next generation of high-performance devices.

**Figure 6:**
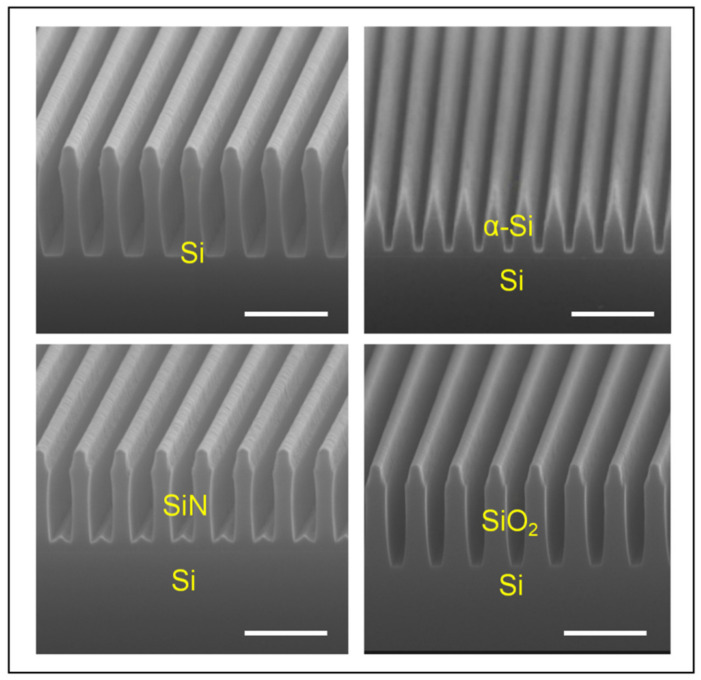
The one-step etching method is suitable for preparing two-hierarchical structures of various materials, such as c-Si, α-Si, SiN_x_, and SiO_2_. Scale bar: 800 nm.

Most importantly, the one-step etching process of two-hierarchical nanostructures can be expanded to a series of materials because the generation of a passivation layer and the shrinking of the mask pattern are not affected by the target materials. Here, Si-based materials including c-Si, α-Si, SiN_x_, and SiO_2_, which play crucial roles in both electronic and photonics devices, are introduced to show the material universality of the developed method. Figure 6 demonstrates the two-hierarchical nanostructure etching by the one-step etching method. The masks are Cr, the linewidth is 200 nm, and the period is 300 nm. All materials are etched under the parameters of ICP power 350 W and RF power 120 W; the gas flows are fixed at SF_6_ 40 sccm, CHF_3_ 10 sccm, and O_2_ 5 sccm; the chamber pressure is set to 10 mTorr; and the temperature is 40 °C. It can be found that although the morphologies of the nanostructures are different, a two-hierarchical configuration is clearly observed. The final etching morphologies of c-Si, SiN_x_, and SiO_2_ are similar, while the upper structure of α-Si etching is sharper, which is related to the specific kind of material. This verifies that one-step etching can be applied to abundant suitable materials and can be used in lots of devices with high performance.

## 4. Conclusions

We have proposed a one-step etching method to directly build up two-hierarchical nanostructures, based on the simultaneous generation of a passivation layer on the side wall and the shrinking effect of the mask pattern during the etching process. Two-hierarchical nanostructures comprising a triangle upper layer and a rectangle lower layer are successfully constructed, showing that the main feature parameters of the nanostructures, such as the angle and height of the triangle in the upper layer, and the height and linewidth of the rectangle in the lower layer, can be tunable over a large range by varying the etching conditions. TiO_2_ two-hierarchical nanostructure arrays with gaps down to 10 nm and a complex mask can be fabricated by this method. Moreover, the one-step etching can be expanded to a series of other materials with important applied value, which ensures the method can be utilized in high-performance devices that need two-hierarchical nanostructures. This work provides a new technology strategy for fabricating two-hierarchical nanostructures in the field of photonic and electronic nanodevices, and further helps the design and realization of related devices with stronger ability and new functions.

## Data Availability

Dataset available on request from the authors.
